# Household beliefs about malaria testing and treatment in Western Kenya: the role of health worker adherence to malaria test results

**DOI:** 10.1186/s12936-017-1993-7

**Published:** 2017-08-22

**Authors:** Indrani Saran, Elisa M. Maffioli, Diana Menya, Wendy Prudhomme O’Meara

**Affiliations:** 1 Duke Global Health Institute, Duke University, 310 Trent Drive, Durham, NC 27701 USA; 2 Department of Economics, Duke University, Durham, USA; 3 School of Public Health, College of Health Sciences, Moi University, Eldoret, Kenya; 4 Duke University Medical Center, Duke University, Durham, USA

**Keywords:** Malaria, Household beliefs, Targeting, Rapid diagnostic tests, Artemisinin-combination therapy, Treatment-seeking behavior, Case management

## Abstract

**Background:**

Although use of malaria diagnostic tests has increased in recent years, health workers often prescribe anti-malarial drugs to individuals who test negative for malaria. This study investigates how health worker adherence to malaria case management guidelines influences individuals’ beliefs about whether their illness was malaria, and their confidence in the effectiveness of artemisinin-based combination therapy (ACT).

**Methods:**

A survey was conducted with 2065 households in Western Kenya about a household member’s treatment actions for a recent febrile illness. The survey also elicited the individual’s (or their caregiver’s) beliefs about the illness and about malaria testing and treatment. Logistic regressions were used to test the association between these beliefs and whether the health worker adhered to malaria testing and treatment guidelines.

**Results:**

Of the 1070 individuals who visited a formal health facility during their illness, 82% were tested for malaria. ACT rates for malaria-positive and negative individuals were 89 and 49%, respectively. Overall, 65% of individuals/caregivers believed that the illness was “very likely” malaria. Individuals/caregivers had higher odds of saying that the illness was “very likely” malaria when the individual was treated with ACT, and this was the case both among individuals not tested for malaria [adjusted odds ratio (AOR) 3.42, 95% confidence interval (CI) [1.65 7.10], P = 0.001] and among individuals tested for malaria, regardless of their test result. In addition, 72% of ACT-takers said the drug was “very likely” effective in treating malaria. However, malaria-negative individuals who were treated with ACT had lower odds of saying that the drugs were “very likely” effective than ACT-takers who were not tested or who tested positive for malaria (AOR 0.29, 95% CI [0.13 0.63], P = 0.002).

**Conclusion:**

Individuals/caregivers were more likely to believe that the illness was malaria when the patient was treated with ACT, regardless of their test result. Moreover, malaria-negative individuals treated with ACT had lower confidence in the drug than other individuals who took ACT. These results suggest that ensuring health worker adherence to malaria case management guidelines will not only improve ACT targeting, but may also increase patient/caregivers’ confidence in malaria testing and treatment.

**Electronic supplementary material:**

The online version of this article (doi:10.1186/s12936-017-1993-7) contains supplementary material, which is available to authorized users.

## Background

Over the past 15 years, global malaria mortality has declined by 48% largely due to expanding coverage of effective malaria control interventions such as insecticide-treated bed nets, indoor-residual spraying, and artemisinin-based combination therapy (ACT) [1, 2]. The health benefits of these tools, however, often depend on people’s decision to use them and to do so appropriately [3, 4]. Understanding individuals’ subjective expectations about their illness, about the accuracy of diagnostic technologies, and about the effectiveness of treatment could provide insight into the uptake and use of medications such as artemisinin-based combinations [5].

Most countries with *Plasmodium falciparum* adopted ACT as the first-line treatment for malaria between 2001 and 2008 [6]. While ACT is very effective in treating the disease, the artemisinin-based combinations are often used to treat febrile illnesses that are not malaria [7, 8]. This may delay appropriate treatment for the illness, waste valuable drugs, and also increases the risk of parasites developing resistance to ACT [9, 10].

The widespread overuse of ACT is partly explained by the fact that many febrile individuals are presumptively treated for malaria based on symptoms and do not receive a malaria diagnostic test [7, 11]. Since 2010, when the World Health Organization recommended that all suspected malaria cases receive a confirmed diagnosis before ACT, testing rates in the public health sector have increased dramatically [12]. However, in many contexts, health workers continue to treat individuals who test negative with anti-malarial drugs [13, 14]. In addition, between 30 and 70% of all anti-malarials are distributed in private pharmacies and drug stores where diagnostic testing is rare [15, 16]. Therefore, there has been growing interest in expanding malaria diagnostic testing to private drug shops [17–20]. This has become increasingly feasible due to the commercial availability of malaria rapid diagnostic tests (RDTs), which have simplified malaria testing and provide very accurate results [21, 22].

In order for testing in private drug shops to improve ACT targeting, individuals who suspect malaria need to both choose to get tested and to treat according to the test result. These decisions are likely influenced not only by the relative costs of testing and treatment, but also by perceptions about the accuracy of a malaria diagnostic test [4, 23]. While qualitative studies have examined community attitudes towards testing, there is little evidence on how people learn about the benefits of malaria testing [24–26]. This study hypothesized that individuals’ experiences at formal health facilities influences their beliefs about malaria testing and treatment. The analysis investigates how health workers’ adherence to malaria case management guidelines is associated with individuals’ (or their caregivers) beliefs about the likelihood their illness was malaria, and their beliefs about the effectiveness of ACT.

## Methods

### Study context and population

The study used data from a survey conducted with 2065 households randomly sampled within 34 community units (which consists of approximately 1000 households each) in two sub-counties in Western Kenya: Bungoma East and Kiminini. The survey, conducted between June and November 2015, served as the baseline for a randomized controlled trial examining the public health impact of expanding malaria testing in communities, described in more detail elsewhere [27].

Malaria is highly endemic in this region with peaks in transmission during the rainy seasons (March through May and October through December) [28]. At the time of data collection, malaria diagnostic testing was primarily available at formal health facilities. In the public sector, malaria diagnostic tests were supposed to be free for children under the age of five but there was a fee for older children and adults (the median reported cost for malaria tests in our survey was 50 Kenyan shillings or approximately USD 0.50). ACT was free at public health facilities and available at subsidized prices at private facilities including informal sector drug shops (an adult dose cost between 100 and 120 Kenyan shillings, equivalent to USD 1–1.20) [29, 30].

### Data

The inclusion criteria for the survey was that at least one member of the household had a fever or malaria-like illness in the past four weeks. Survey respondents were individuals who were 18 years or older or the sick individual’s primary caregiver if he/she was younger than 18. The survey collected demographic information about the household, the respondent, and the sick individual.

Individuals/caregivers were asked if they sought any treatment for the illness, the primary and secondary treatment sources, whether a malaria diagnostic test was performed, the test result, and whether any drugs were taken for the illness.

Several questions in the survey were designed to assess respondents’ beliefs about testing and treatment. These beliefs were elicited using a five-point Likert scale from “very unlikely” to “very likely”. In order to measure confidence in malaria testing, respondents were asked about the likelihood that a malaria test result is correct if a febrile patient tests positive and, separately, if a febrile patient tests negative. Individuals who were treated with ACT were asked about the effectiveness of ACT in treating malaria. All respondents were also asked about the likelihood that the individual’s illness was malaria.

The surveys were administered verbally by trained interviewers in either Kiswahili or English according to the preference of the respondent. Most questions were open-ended, however the survey included a set of pre-coded answers as well as the option “other” for responses that the interviewer believed did not fit into any of the previously coded answer choices. In the analysis, responses provided in the “other” category were examined and, if appropriate, re-categorized into one of the previously coded responses.

### Analytical approach

Since the goal was to investigate how respondents’ beliefs about testing and treatment were associated with health workers’ adherence to malaria case management guidelines, all analyses were limited to individuals who, during the course of the illness, had visited either a public or private health facility. The analysis first examined whether the sick individual was tested for malaria and treated according to the test result. The second step was to test the association between individuals/caregivers’ beliefs about malaria likelihood and the sick individuals’ test status and ACT use. The last analysis focused on the variation in ACT-takers’ confidence in ACT by the sick individuals’ test status.

Health workers’ adherence to case management guidelines were based on respondents’ reports of whether the sick individual was tested for malaria, their test result, and whether they were treated with ACT. A sick individual was defined as having been treated with ACT if the respondent said they took an ACT medicine or the brand name of the drug they took indicated it was an artemisinin-based combination (such as “Coartem” or “Lumartem”). In order to verify these self-reports on testing and treatment, the interviewer asked to see the record for the test result if it was available and also asked to see the packaging of the anti-malarial that was taken. The robustness of the main results was tested by limiting the sample only to individuals who had a record for their test result and for whom the ACT package was observed.

Individual/caregiver beliefs about malaria likelihood were split into two categories: those who believed that it was “very likely” that the patient’s illness was malaria and everybody else (“very likely” was the most common response). All other measures of respondent beliefs were similarly dichotomized.

The results section presents graphs to show how ACT use, and confidence in ACT, varies with the sick individual’s test status (not tested, tested negative, tested positive). Logistic regressions were used to test whether the graphical relationships were statistically significant and to control for other factors that evidence from the literature suggests might influence the outcomes and might also be associated with an individual’s test status [31–35]. These factors included the sick individual’s age and gender, the education level of the respondent, the wealth quintile of the household, and the distance of the household to the closest health facility (the models showing the coefficients on the control variables are presented in Additional files 1, 2).

A respondent was defined as having some primary education if they completed part of, or all of, primary school (but had no further education) and some secondary education if they had any education beyond primary school. Households were assigned to wealth quintiles using a polychoric principal component analysis of housing characteristics and household ownership of durable assets and farm animals [36]. Household distance to the nearest government health facility was based on self-reported travel time (in minutes).

In all regressions, standard errors were adjusted for clustering at the community unit level (there were 34 community units). Analyses were conducted using Stata/SE version 14 (StataCorp, College Station, TX, USA) [37].

## Results

### Sample characteristics

A total of 3866 households were visited, 2065 (53%) of which met the survey inclusion criteria of having a household member who had a fever or malaria-like illness in the four weeks prior to the visit. Among the 2065 households included in the survey, 2007 (97%) had febrile individuals who had taken an action for their illness (this included having taken medicines at home). Table 1 presents summary statistics on the analysis sample—the 53% of sick individuals (1070/2007) who had visited a formal health facility. Approximately 28% of individuals were under the age of five. Survey respondents were on average 40 years old, 85% were female, and 96% had some education. While 91% of households owned a mobile phone, only 20% had electricity.

**Table 1:** Summary statistics of demographic characteristics and treatment behavior

	Individuals who ever visited formal health facility (N = 1070)		
	Mean ± SD	N (%)	Total non-missing observations
*A. Characteristics of patient*			
Age	19 ± 19		1070
Patient under 5		295 (28)	1070
Female		650 (61)	1070
*B. Characteristics of respondent*			
Age	40 ± 14		1070
Female		907 (85)	1070
No education		44 (4)	1070
Some primary education		592 (55)	1070
Some secondary education		434 (41)	1070
*C. Characteristics of household*			
Has electricity		212 (20)	1069
Owns mobile phone		978 (91)	1069
Owns land		974 (91)	1069
Number of household members	6.6 ± 2.6		1070
Time to nearest health facility (min)	26 ± 15		1049
*D. Treatment*-*seeking for febrile illness*			
Took more than one action for illness		653 (61)	1070
Ever visited public sector		778 (73)	1070
Ever visited private clinic		324 (30)	1070
Ever visited drug shop/pharmacy		384 (36)	1070
*E. Malaria testing and treatment*			
Tested for malaria		875 (82)	1067
Among those tested			
Tested with microscopy		448 (51)	875
Tested with RDT		340 (39)	875
Don’t know/don’t remember test type		87 (10)	875
Tested at formal health facility		871 (100)	875
Tested positive		720 (83)	871
Record observed for test result		474 (54)	875
Took ACT		853 (80)	1069
ACT packaging observed		390 (46)	853
Took other anti-malarial drug		215 (20)	1069
Took any anti-malarial drug (including ACT)		912 (85)	1069

Individuals who visited each treatment location during their illness (public sector, private sector, drug shop/pharmacy) may have also taken other treatment actions

More than 60% of individuals had taken multiple actions for their illness. Approximately 73% of individuals had sought care in a public health facility, 30% had visited a private clinic and 36% had visited a drug shop or pharmacy. Among the 82% of individuals who were tested for malaria, 83% reported a positive test result. Consistent with testing being primarily available at public and private health facilities, more than 99% of individuals who were tested for malaria reported being tested at a formal health facility.

Overall, 80% of sick individuals who visited a health facility were treated with ACT and 85% were treated with any type of anti-malarial drug (including artemisinin-based combinations). Among those who were tested for malaria, 54% had a record for their test result that was observed by the interviewer. For those who reported taking ACT, 46% had the drug packaging available and the type of drug was verified by the interviewer.

Table 2 shows the distribution of responses to the Likert-scale beliefs questions about the illness, about testing and about treatment. When individuals/caregivers were asked about the illness, 65% said it was “very likely” that it was malaria. Regarding malaria testing, 84% of respondents said that a positive malaria test was “very likely” to be correct but only 30% said the same about a negative malaria test. Among ACT-takers, 72% believed that the drug was “very likely” to be effective in treating malaria.

**Table 2:** Summary of responses to Likert scale questions on beliefs

	Response to question N (%)					Observations
	Very unlikely	Unlikely	50–50	Likely	Very likely	
1. How likely is it that the illness was malaria?	11 (1.1)	31 (3.0)	100 (9.5)	223 (21)	687 (65)	1052
2. How likely do you think the malaria drug you take/took is/was effective?	17 (2.1)	23 (2.8)	41 (5)	148 (18)	594 (72)	823
3. If you have fever and your malaria test is negative, how likely is it that the test is correct	267 (26)	124 (12)	141 (14)	205 (20)	309 (30)	1046
4. If you have fever and your malaria test is positive, how likely is it that the test is correct	12 (1.1)	1 (0.1)	15 (1.4)	145 (14)	885 (84)	1058

Responses for Question 2 are limited to sick individuals who were treated with ACT

### Adherence to malaria case management guidelines

**Fig. 1:**
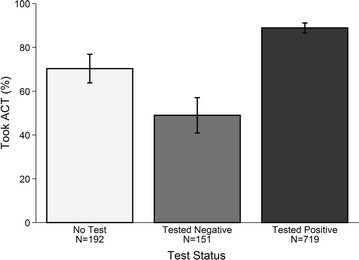
ACT use by individual’s test status. The proportion of sick individuals who visited a formal health facility (N = 1070) who were treated with an ACT by whether they were tested for malaria and by their test result. *Error bars* indicate 95% confidence intervals. 8 individuals were missing information on their test status or on whether they took an ACT

Figure 1 presents testing and treatment rates for individuals who visited a formal health facility. Approximately 18% (192/1062) of individuals were not tested for malaria and, among these individuals, 70% were treated with an artemisinin-based combination. ACT rates for malaria-positive individuals and malaria-negative individuals were 89 and 49%, respectively. ACT use was associated with the individuals’ test status: when compared to individuals not tested for malaria, individuals who tested positive had 3.41 times the adjusted odds of being treated with an ACT (95% CI [2.23 5.21], P < 0.001), while the odds of receiving ACT was halved among individuals who tested negative for malaria (adjusted odds ratio (AOR) 0.45, 95% CI [0.28 0.71], P = 0.001) (Table 3). The proportions of malaria-positive and malaria-negative individuals treated with ACT were similar irrespective of whether the patient was tested using microscopy or an RDT (Additional file 3).

**Table 3:** Association between test status, ACT, and confidence in ACT

	Outcome: odds of taking ACT		Outcome: odds respondent believed ACT “very likely” effective in treating malaria	
	OR	AOR	OR	AOR
	(1)	(2)	(3)	(4)
A. Tested positive for malaria	3.37** [2.21, 5.15]	3.41** [2.23, 5.21]	1.35 [0.76, 2.42]	1.25 [0.63, 2.50]
B. Tested negative for malaria	0.41** [0.27, 0.62]	0.45** [0.28, 0.71]	0.33** [0.17, 0.65]	0.29** [0.13, 0.63]
C. Not tested for malaria	Ref. Group	Ref. Group	Ref. Group	Ref. Group
Includes controls		X		X
Mean of outcome in reference group	0.7	0.7	0.7	0.7
P value: (A = B)	0	0	0	0
Number of observations	1062	1041	818	806

Table shows logistic regression results of the association between test status and ACT use (columns 1 and 2), and beliefs about ACT effectiveness (columns 3 and 4). Columns 3 and 4 are limited to individuals who were treated with ACT. The controls in Columns 2 and 4 include the following: the wealth of the household (defined as the first component from a principal component analysis of household characteristics and assets), the education level of the respondent (no education, some primary education, or some secondary education), the sick individual’s age and gender, and the time it takes for the household to travel to the nearest health facility. All coefficients are expressed in terms of odds ratios and 95% confidence intervals are in brackets. Standard errors are adjusted for clustering by community unit. ** P < 0.01

### Beliefs about malaria likelihood

Table 4 displays the association between individuals/caregivers’ beliefs about malaria likelihood and individuals’ testing and treatment status. Approximately 35% of individuals who were not tested for malaria and not treated with an ACT (the reference group) said their illness was “very likely” to have been malaria. Individuals with a positive test, who were not treated with an ACT, had significantly higher odds of believing their illness was “very likely” malaria compared to those not tested and not treated with an ACT (AOR 2.75, 95% CI [1.41 5.38], P = 0.003) while individuals testing negative who were not treated with ACT had significantly lower odds of saying the same about their illness (AOR 0.37 95% CI [0.18 0.73], P = 0.004).

**Table 4:** Association between malaria beliefs, testing and ACT use

	Outcome: respondent said illness was “very likely” malaria	
	OR	AOR
A. Tested positive for malaria, not treated with ACT	2.83** [1.45, 5.53]	2.75** [1.41, 5.38]
B. Tested negative for malaria, not treated with ACT	0.42** [0.22, 0.81]	0.37** [0.18, 0.73]
C. Not tested for malaria, treated with ACT	3.34** [1.63,6.85]	3.42** [1.65,7.10]
D. Tested positive for malaria, treated with ACT	6.41** [3.63,11.31]	6.32** [3.62,11.01]
E. Tested negative for malaria, treated with ACT	1.24 [0.63, 2.48]	1.18 [0.62, 2.25]
F. Not tested for malaria, not treated with ACT	Ref. Group	Ref. Group
Includes controls		X
P value: A = D	0.001	0.003
P value: B = E	0	0
Proportion believed illness “very likely” malaria in reference group	0.346	0.346
Number of observations	1046	1025

Table shows logistic regression results of the association between both test status and ACT use and beliefs about malaria likelihood. The controls in column 2 include the following: the wealth of the household (defined as the first component from a principal component analysis of household characteristics and assets), the education level of the respondent (no education, some primary education, or some secondary education), the sick individual’s age and gender, and the time it takes for the household to travel to the nearest health facility. All coefficients are expressed in terms of odds ratios and confidence intervals are in brackets. Standard errors are adjusted for clustering by community unit. ** P < 0.01

In addition, regardless of test status, individuals/caregivers were more likely to say that the illness was “very likely” malaria when the sick individual was treated with ACT. Among individuals not tested for malaria, ACT use increased the adjusted odds of individuals/caregivers saying that the illness was “very likely” malaria by a factor of 3.42 (95% CI [1.65 7.10], P = 0.001). ACT use also increased the odds of the respondent saying that the illness was “very likely” malaria among sick individuals who tested positive for malaria and sick individuals who tested negative for malaria (Table 4).

### Beliefs about ACT effectiveness

**Fig. 2:**
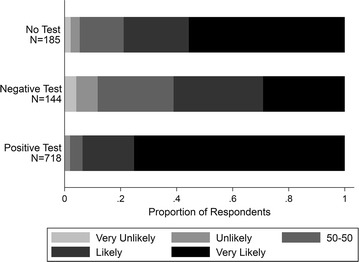
Respondents’ beliefs about ACT effectiveness by test status. Distribution of respondents’ beliefs about the effectiveness of ACT by whether they were tested for malaria and by their test result. Sample is limited to individuals who ever visited a health facility and were treated with an ACT (N = 853). Responses were based on a 5-point Likert Scale ranging from “very unlikely” to “very likely”. 35 patients were missing information on either their test status or beliefs about ACT effectiveness

Figure 2 shows how individual/caregivers’ beliefs about ACT effectiveness varied by health workers adherence to malaria treatment guidelines. The sample is limited to individuals who ever visited a formal health facility and were treated with ACT. Approximately 70% of individuals who were not tested for malaria said that ACT was “very likely” effective in treating malaria. While there was no statistically significant difference in individual/caregivers’ confidence in ACT when individuals tested positive for malaria (AOR 1.25, 95% CI [0.63 2.50], P = 0.522), individuals who tested negative for malaria and were treated with ACT had lower odds of saying that ACT were “very likely” effective in treating malaria (AOR 0.29, 95% CI [0.13 0.63], P = 0.002) (Table 3).

## Discussion

The benefits of expanding access to malaria testing will only be realized if suspected malaria cases receive a confirmed diagnosis before treatment. This study has three main results. First, most (>80%) individuals visiting a formal health facility in this context were tested for malaria, and ACT use was associated with the diagnostic test result. Second, individuals’/caregivers’ beliefs about whether their illness was malaria were independently associated with both the diagnostic test result and also with whether the sick individual was treated with ACT. Third, malaria-negative individuals who were incorrectly treated with ACT had lower confidence in the drug than other individuals treated with ACT.

Although this study finds that malaria testing is being commonly used in formal health facilities, and high rates of ACT use for malaria-positive individuals, approximately 50% of individuals who tested negative for malaria were still treated with an ACT. This suggests that either health workers lack confidence in the negative test result, or that they do not have the necessary skills, training or support to confidently provide an alternative diagnosis. This result corresponds with evidence from other clinical contexts of low adherence to a negative test result [13, 14]. Moreover, as in other studies, individuals and caregivers also expressed greater confidence in a malaria positive test result than a negative test result [26, 38], perhaps because a positive result confirms individuals’ expectations of having malaria [39, 40]. In the case of RDTs, adherence to the test may increase over time as both health workers and individuals gain exposure to the technology [41, 42]. In some contexts, increased training of health workers, or targeting individuals’ malaria knowledge and perceptions has been shown to improve test adherence [43–46].

This is one of a few studies to examine how beliefs about malaria are associated with diagnostic testing and with treatment [23, 47, 48]. The results show that individuals’/caregivers’ beliefs about malaria likelihood are associated with the malaria test result, though this is likely to be a combination of differences in beliefs prior to testing and updating of beliefs based on the test result. However, the results also suggest that, regardless of test status, individuals/caregivers were more likely to believe that the illness was malaria when the sick individual was treated with ACT. This may be because being prescribed an ACT is perceived as synonymous with a diagnosis [49] or because treatment reinforces beliefs that the illness was malaria (particularly if the illness resolved following treatment).

Lastly, this study finds that individuals who tested negative and were treated with ACT had lower confidence in effectiveness of the drug than other individuals who were treated with ACT. This suggests that individuals’ whose illness did not resolve with ACT concluded that the drugs were not effective in treating malaria. These results coincide with evidence from Tanzania, which suggests that high rates of malaria mis-diagnosis hampers patient learning about ACT effectiveness [50]. It is also possible that respondents interpreted the question about drug effectiveness specifically in terms of treating the current illness (and not malaria more generally) and may have recognized that the ACT they took was not effective for malaria-negative individuals because their illness was not malaria. However, 75% of respondents for malaria-negative individuals who were treated with an ACT still believed that it was “likely” or “very likely” that the illness was malaria.

There are several limitations to this study. First, although individuals were visiting a health facility, individual/caregiver confidence in testing may have influenced whether the sick individual was tested for malaria and adherence to the test result. However, there are several pieces of evidence to suggest that the testing and treatment decisions were made primarily by the health worker. First, regardless of test status, more than 84% of individuals obtained ACT either at the health facility or at a pharmacy with a prescription (Additional file 4). This suggests that, in most cases, ACT was prescribed by the health worker. Second, there is no evidence that individuals’/caregivers’ confidence in a malaria test result is associated with the probability of being tested (Additional file 5) or with adherence to a negative test result (Additional file 6). It is important to note, however, that confidence in the test was measured after individuals had already sought care for their illness, and testing and treatment may have affected confidence in testing. The survey did not collect information on individual/caregiver beliefs prior to seeking care and, therefore, it is not possible to examine how these beliefs are associated with testing and treatment.

A second limitation is that the study relies on individuals/caregivers’ self-reports of their malaria test result, and individuals treated with ACT may have assumed that they tested positive for malaria. However, when the analyses are limited to the 414 (39%) individuals who had a record for their test result (if they reported being tested), and who had the packaging of their ACT observed (if they reported taking ACT) results are similar though the confidence intervals are wide due to the smaller sample size (Additional files 7, 8).

Third, since beliefs about ACT effectiveness were only elicited for individuals treated with ACT, it is not possible to test how confidence in the drug is associated with whether an individual was treated with ACT. They survey also did not collect data on the length of individuals’ illness to examine how this correlates with individuals’/caregivers’ beliefs about ACT effectiveness.

Fourth, it is possible that people’s definition of malaria is broader than the biomedical definition of an infection that can be detected by a test and treated with ACT [51]. As a result, a person’s belief about whether the sick individual’s illness was malaria may not necessarily reflect his/her confidence in the test result. However, in a pilot study conducted in the same area, respondents demonstrated awareness of the transmissibility of malaria by mosquitoes and the correct treatment of the illness, suggesting an understanding of the biomedical basis of the disease (unpublished observations).

Lastly, the way people’s beliefs about malaria likelihood and beliefs about ACT effectiveness were dichotomized (“very likely” compared to all others) means that the analysis focuses on the degree to which respondents are fully confident in their response or have some uncertainty. Although the results show that respondents’ certainty about whether the illness was malaria and beliefs about ACT effectiveness vary by their testing and treatment outcomes, it is not possible to say to what extent these differences in beliefs may influence individuals’ future malaria treatment decisions.

## Conclusion

The results of this study suggest that health worker non-adherence to negative malaria test results has important implications for individuals’ beliefs about their illness and about treatment. Thus, increasing health workers’ adherence to malaria treatment guidelines—for example by reinforcing their trust in the test or by offering training and support on management of non-malarial febrile illnesses—would not only directly improve ACT targeting, but may also raise people’s confidence in testing and treatment. This could affect whether individuals/caregivers choose to get tested for future illnesses and adhere to the test result, a growing concern as diagnostic testing is expanded to the informal private sector. Further research is needed to understand how individual beliefs about testing and treatment at the end of one illness episode affect treatment behaviors for future illnesses.

## Additional files

**Additional file 1.** Associations between Test Status, ACT Use and Confidence in ACT—Full Model. Table shows logistic regression results of the association between test status and ACT use (Columns 1 and 2) and beliefs about ACT effectiveness (Columns 3 and 4) including the coefficients on the control variables.

**Additional file 2.** Associations between Malaria Beliefs, Testing, and ACT Use—Full Model. Table shows logistic regression results of the association between both test status and ACT use and beliefs about malaria likelihood for individuals including the coefficients on the control variables.

**Additional file 3.** Adherence to Test Result by Test Type. Proportion of individuals who took an ACT by test result and by whether they were tested using microscopy or RDT.

**Additional file 4.** Source of ACT drugs by Test Status. Percentage of individuals who received ACT from each treatment source by their test status.

**Additional file 5.** Probability of Being Tested by Respondents’ Confidence in Testing. Figure shows the proportion of individuals who were tested by respondents’ beliefs about the likelihood that a positive test result is correct (Panel A) and beliefs about the likelihood that a negative test result is correct (Panel B).

**Additional file 6.** Probability of ACT Use by Respondents’ Confidence in Testing. Figure shows the proportion of individuals treated with an ACT separately by whether they tested positive (Panel A) or tested negative (Panel B) and by respondents’ beliefs about the likelihood that such a test result is correct.

**Additional file 7.** Associations between Test Status, ACT Use, and Confidence in ACT- Individuals with Test Record and ACT Packaging Only. Table shows logistic regression results of the association between test status and ACT use (Columns 1 and 2) and beliefs about ACT effectiveness (Columns 3 and 4) for individuals who had a record of their test result and ACT-takers who showed the packaging of their drug.

**Additional file 8.** Associations between Malaria Beliefs, Testing, and ACT Use - Individuals with Test Record and ACT Packaging Only. Table shows logistic regression results of the association between both test status and ACT use and beliefs about malaria likelihood for individuals who had a record for their test result and ACT-takers who showed the packaging of their drug.
