# Supplementary material for: Household beliefs about malaria testing and treatment in Western Kenya: the role of health worker adherence to malaria test results
Source: Malar J. 2017 Aug 22;16:349. doi: 10.1186/s12936-017-1993-7 (PMC5568326; doi:10.1186/s12936-017-1993-7)
Supplement: Supplementary file 1 — Additional file 1. Associations between Test Status, ACT Use and Confidence in ACT—Full Model. Table shows logistic regression results of the association between test status and ACT use (Columns 1 and 2) and beliefs about ACT effectiveness (Columns 3 and 4) including the coefficients on the control variables. [file 12936_2017_1993_MOESM1_ESM.docx]

**Associations between Test Status, ACT Use and Confidence in ACT- Full Model**

|  | Outcome: Odds of Taking ACT | |  |  | Outcome: Respondent Believed ACT "Very Likely" Effective in Treating Malaria | |
| --- | --- | --- | --- | --- | --- | --- |
|  | OR | AOR |  |  | OR | AOR |
|  | (1) | (2) |  |  | (3) | (4) |
| A. Tested Positive for Malaria | 3.37** | 3.41** |  |  | 1.35 | 1.25 |
|  | [2.21,5.15] | [2.23,5.21] |  |  | [0.76,2.42] | [0.63,2.50] |
|  |  |  |  |  |  |  |
| B. Tested Negative for Malaria | 0.41** | 0.45** |  |  | 0.33** | 0.29** |
|  | [0.27,0.62] | [0.28,0.71] |  |  | [0.17,0.65] | [0.13,0.63] |
|  |  |  |  |  |  |  |
| C. Not Tested for Malaria | Ref. Group | Ref. Group |  |  | Ref. Group | Ref. Group |
|  |  |  |  |  |  |  |
|  |  |  |  |  |  |  |
| D. Wealth Quintile 1 (Poorest) |  | Ref. Group |  |  |  | Ref. Group |
|  |  |  |  |  |  |  |
|  |  |  |  |  |  |  |
| E. Wealth Quintile 2 |  | 1.18 |  |  |  | 0.98 |
|  |  | [0.63,2.19] |  |  |  | [0.56,1.74] |
|  |  |  |  |  |  |  |
| F. Wealth Quintile 3 |  | 1.05 |  |  |  | 0.93 |
|  |  | [0.50,2.20] |  |  |  |  |
|  |  |  |  |  |  |  |
| G. Wealth Quintile 4 |  | 0.93 |  |  |  | 1.19 |
|  |  | [0.56,1.54] |  |  |  | [0.64,2.19] |
|  |  |  |  |  |  |  |
| H. Wealth Quintile 5 (Richest) |  | 0.71 |  |  |  | 1.27 |
|  |  | [0.47,1.09] |  |  |  | [0.64,2.53] |
|  |  |  |  |  |  |  |
| I. Respondent Has No Education |  | Ref. Group |  |  |  | Ref. Group |
|  |  |  |  |  |  |  |
|  |  |  |  |  |  |  |
| J. Respondent Has Some Primary Education |  | 1.52 |  |  |  | 0.48 |
|  |  | [0.67,3.43] |  |  |  | [0.17,1.30] |
|  |  |  |  |  |  |  |
| H. Respondent Has Some Secondary Education |  | 2.17 |  |  |  | 0.57 |
|  |  | [0.96,4.87] |  |  |  | [0.21,1.54] |
|  |  |  |  |  |  |  |
| I. Age of Sick Individual |  | 1 |  |  |  | 0.99* |
|  |  | [0.99,1.00] |  |  |  | [0.98,1.00] |
|  |  |  |  |  |  |  |
| J. Sick Individual is Female |  | 0.79 |  |  |  | 1.08 |
|  |  | [0.52,1.19] |  |  |  | [0.84,1.39] |
|  |  |  |  |  |  |  |
| K. Time to Nearest Health Facility (Minutes) |  | 1 |  |  |  | 1 |
|  |  | [0.99,1.02] |  |  |  | [0.99,1.01] |
|  |  |  |  |  |  |  |
| Mean of Outcome in Reference Group | 0.7 | 0.7 |  |  | 0.7 | 0.7 |
| P value: (A=B) | 0 | 0 |  |  | 0 | 0 |
| Number of Obs | 1062 | 1041 |  |  | 818 | 806 |

Notes: Table shows logistic regression results of the association between test status and ACT use (Columns 1 and 2) and beliefs about ACT effectiveness (Columns 3 and 4). Columns 3 and 4 are limited to individuals who were treated with an ACT. All coefficients are expressed in terms of odds ratios and 95 % confidence intervals are in brackets. Standard errors are adjusted for clustering by community unit. *p<0.05, **p<0.01
