# Supplementary material for: Household beliefs about malaria testing and treatment in Western Kenya: the role of health worker adherence to malaria test results
Source: Malar J. 2017 Aug 22;16:349. doi: 10.1186/s12936-017-1993-7 (PMC5568326; doi:10.1186/s12936-017-1993-7)
Supplement: Supplementary file 2 — Additional file 2. Associations between Malaria Beliefs, Testing, and ACT Use—Full Model. Table shows logistic regression results of the association between both test status and ACT use and beliefs about malaria likelihood for individuals including the coefficients on the control variables. [file 12936_2017_1993_MOESM2_ESM.docx]

**Associations between Malaria Beliefs, Testing and ACT Use – Full Model**

|  | Outcome: Respondent Said Illness Was "Very Likely" Malaria | |
| --- | --- | --- |
|  | OR | AOR |
|  | (1) | (2) |
| A. Tested Positive for Malaria, Not Treated with ACT | 2.83** | 2.75** |
|  | [1.45,5.53] | [1.41,5.38] |
|  |  |  |
| B. Tested Negative for Malaria, Not Treated with ACT | 0.42** | 0.37** |
|  | [0.22,0.81] | [0.18,0.73] |
|  |  |  |
| C. Not Tested for Malaria, Treated with ACT | 3.34** | 3.42** |
|  | [1.63,6.85] | [1.65,7.10] |
|  |  |  |
| D. Tested Positive for Malaria, Treated with ACT | 6.41** | 6.32** |
|  | [3.63,11.31] | [3.62,11.01] |
|  |  |  |
| E. Tested Negative for Malaria, Treated with ACT | 1.24 | 1.18 |
|  | [0.63,2.48] | [0.62,2.25] |
|  |  |  |
| F. Not Tested for Malaria, Not Treated with ACT | Ref. Group | Ref. Group |
|  |  |  |
|  |  |  |
| G. Wealth Quintile 1 (Poorest) |  | Ref. Group |
|  |  |  |
|  |  |  |
| H. Wealth Quintile 2 |  | 0.7 |
|  |  | [0.41,1.20] |
|  |  |  |
| I. Wealth Quintile 3 |  | 0.66 |
|  |  | [0.38,1.16] |
|  |  |  |
| J. Wealth Quintile 4 |  | 0.74 |
|  |  | [0.41,1.33] |
|  |  |  |
| K. Wealth Quintile 5 (Richest) |  | 0.79 |
|  |  | [0.48,1.30] |
|  |  |  |
| L. Respondent Has No Education |  | Ref. Group |
|  |  |  |
|  |  |  |
| M. Respondent Has Some Primary Education |  | 0.55* |
|  |  | [0.31,0.99] |
|  |  |  |
| N. Respondent Has Some Secondary Education |  | 0.69 |
|  |  | [0.35,1.34] |
|  |  |  |
| O. Age of Sick Individual |  | 0.99** |
|  |  | [0.99,1.00] |
|  |  |  |
| P. Sick Individual is Female |  | 1.01 |
|  |  | [0.75,1.35] |
|  |  |  |
| Q. Time to Nearest Health Facility (Minutes) |  | 1 |
|  |  | [0.99,1.01] |
|  |  |  |
| P-value: A=D | 0.001 | 0.003 |
| P-value: B=E | 0 | 0 |
| Mean Proportion Believed Illness "Very Likely" Malaria in Reference Group | 0.346 | 0.346 |
| Number of Obs | 1046 | 1025 |

Notes: Table shows logistic regression results of the association between both test status and ACT use and beliefs about malaria likelihood. All coefficients are expressed in terms of odds ratios and confidence intervals are in brackets. Standard errors are adjusted for clustering by community unit. *p<0.05, **p<0.01
