# Supplementary material for: Household beliefs about malaria testing and treatment in Western Kenya: the role of health worker adherence to malaria test results
Source: Malar J. 2017 Aug 22;16:349. doi: 10.1186/s12936-017-1993-7 (PMC5568326; doi:10.1186/s12936-017-1993-7)
Supplement: Supplementary file 3 — Additional file 3. Adherence to Test Result by Test Type. Proportion of individuals who took an ACT by test result and by whether they were tested using microscopy or RDT. [file 12936_2017_1993_MOESM3_ESM.docx]

**Adherence to Test Result by Test Type**

|  | Proportion Treated with an ACT | |
| --- | --- | --- |
|  | Tested Negative | Tested Positive |
| Microscopy | 0.57 | 0.89 |
| RDT | 0.44 | 0.90 |
|  |  |  |
| Difference | -0.13 | 0.02 |
| P-value | 0.15 | 0.45 |
| N | 135 | 649 |

Notes: Proportion of individuals who took an ACT by test result and by whether they were tested using microscopy or RDT. P-value is from a t-test of the difference in the proportion of individuals who took an ACT for each test result by type of test.
