# Supplementary material for: Household beliefs about malaria testing and treatment in Western Kenya: the role of health worker adherence to malaria test results
Source: Malar J. 2017 Aug 22;16:349. doi: 10.1186/s12936-017-1993-7 (PMC5568326; doi:10.1186/s12936-017-1993-7)
Supplement: Supplementary file 4 — Additional file 4. Source of ACT drugs by Test Status. Percentage of individuals who received ACT from each treatment source by their test status. [file 12936_2017_1993_MOESM4_ESM.docx]

**Source of ACT drugs by Test Status**

|  | Not Tested | Tested Negative | Tested Positive |
| --- | --- | --- | --- |
| Home | 0.8 | 1.4 | 0.2 |
| Pharmacy With Prescription | 15.0 | 11.0 | 21.3 |
| Pharmacy Without Prescription | 13.5 | 13.7 | 4.1 |
| Private Facility | 46.6 | 9.6 | 19.1 |
| Government Facility | 22.6 | 63.0 | 54.1 |
| General Store | 0.8 | 0.0 | 0.3 |
| Other | 0.8 | 1.4 | 0.8 |
|  |  |  |  |
| Observations | 133 | 73 | 628 |

Notes: Percentage of individuals who received ACTs from each treatment source by their test status. Sample is limited to those who visited a health facility and were treated with ACTs (N=853). 19 individuals were missing information on either their test status or where they obtained the ACTs.
