# Supplementary material for: Household beliefs about malaria testing and treatment in Western Kenya: the role of health worker adherence to malaria test results
Source: Malar J. 2017 Aug 22;16:349. doi: 10.1186/s12936-017-1993-7 (PMC5568326; doi:10.1186/s12936-017-1993-7)
Supplement: Supplementary file 5 — Additional file 5. Probability of Being Tested by Respondents’ Confidence in Testing. Figure shows the proportion of individuals who were tested by respondents’ beliefs about the likelihood that a positive test result is correct (Panel A) and beliefs about the likelihood that a negative test result is correct (Panel B). [file 12936_2017_1993_MOESM5_ESM.docx]

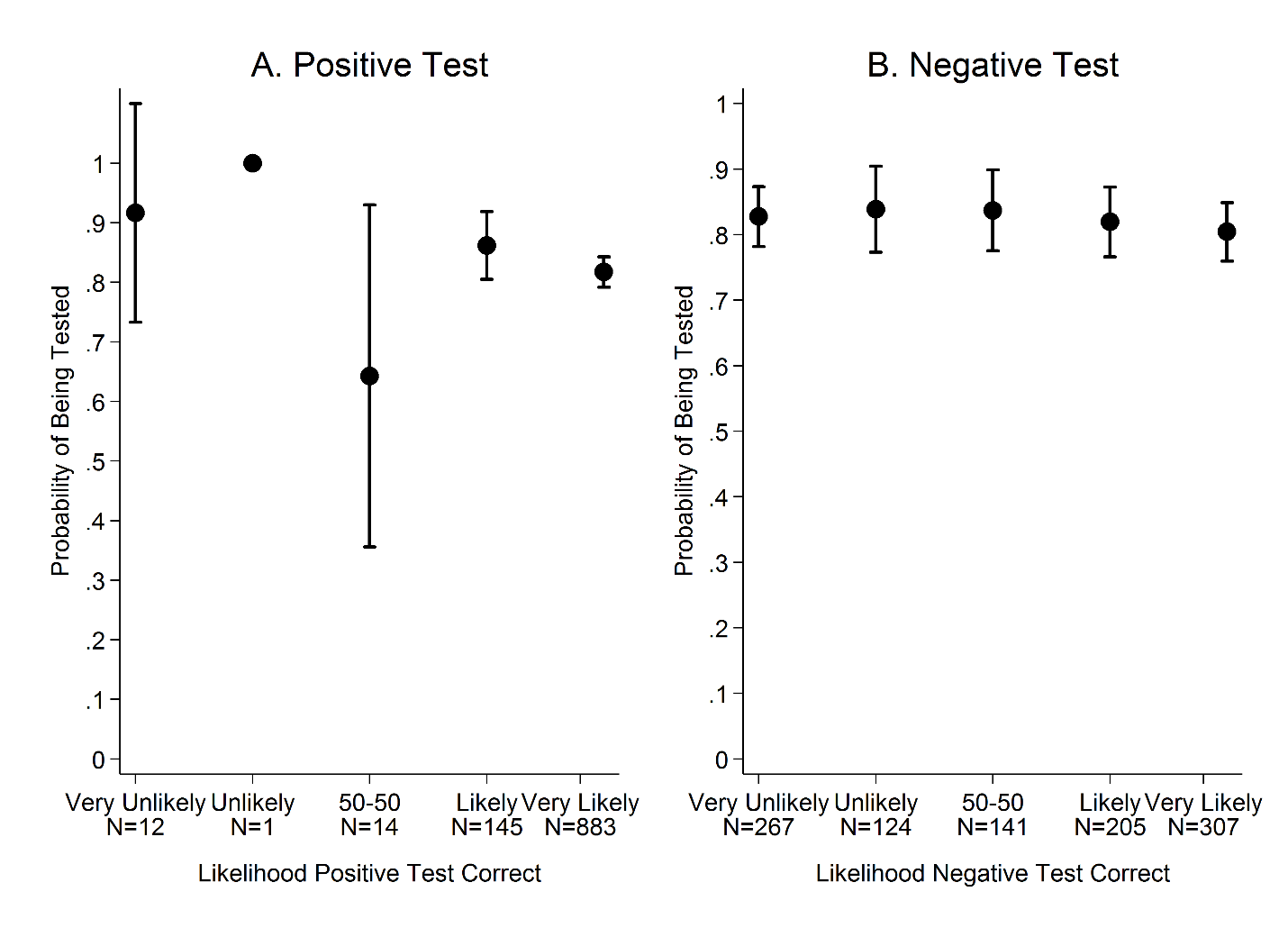


**Probability of Being Tested by Respondents’ Confidence in Testing.** Figure shows the proportion of individuals who were tested by respondents’ beliefs about the likelihood that a positive test result is correct (Panel A) and beliefs about the likelihood that a negative test result is correct (Panel B). Beliefs about confidence in test were elicited *after* testing and treatment. Sample is limited to those who ever visited a health facility (N=1070). 3 individuals were missing information on whether they were tested for malaria, and an additional 12 and 23 individuals were missing information on beliefs about a positive and negative test respectively.
