# Supplementary material for: Household beliefs about malaria testing and treatment in Western Kenya: the role of health worker adherence to malaria test results
Source: Malar J. 2017 Aug 22;16:349. doi: 10.1186/s12936-017-1993-7 (PMC5568326; doi:10.1186/s12936-017-1993-7)
Supplement: Supplementary file 6 — Additional file 6. Probability of ACT Use by Respondents’ Confidence in Testing. Figure shows the proportion of individuals treated with an ACT separately by whether they tested positive (Panel A) or tested negative (Panel B) and by respondents’ beliefs about the likelihood that such a test result is correct. [file 12936_2017_1993_MOESM6_ESM.docx]

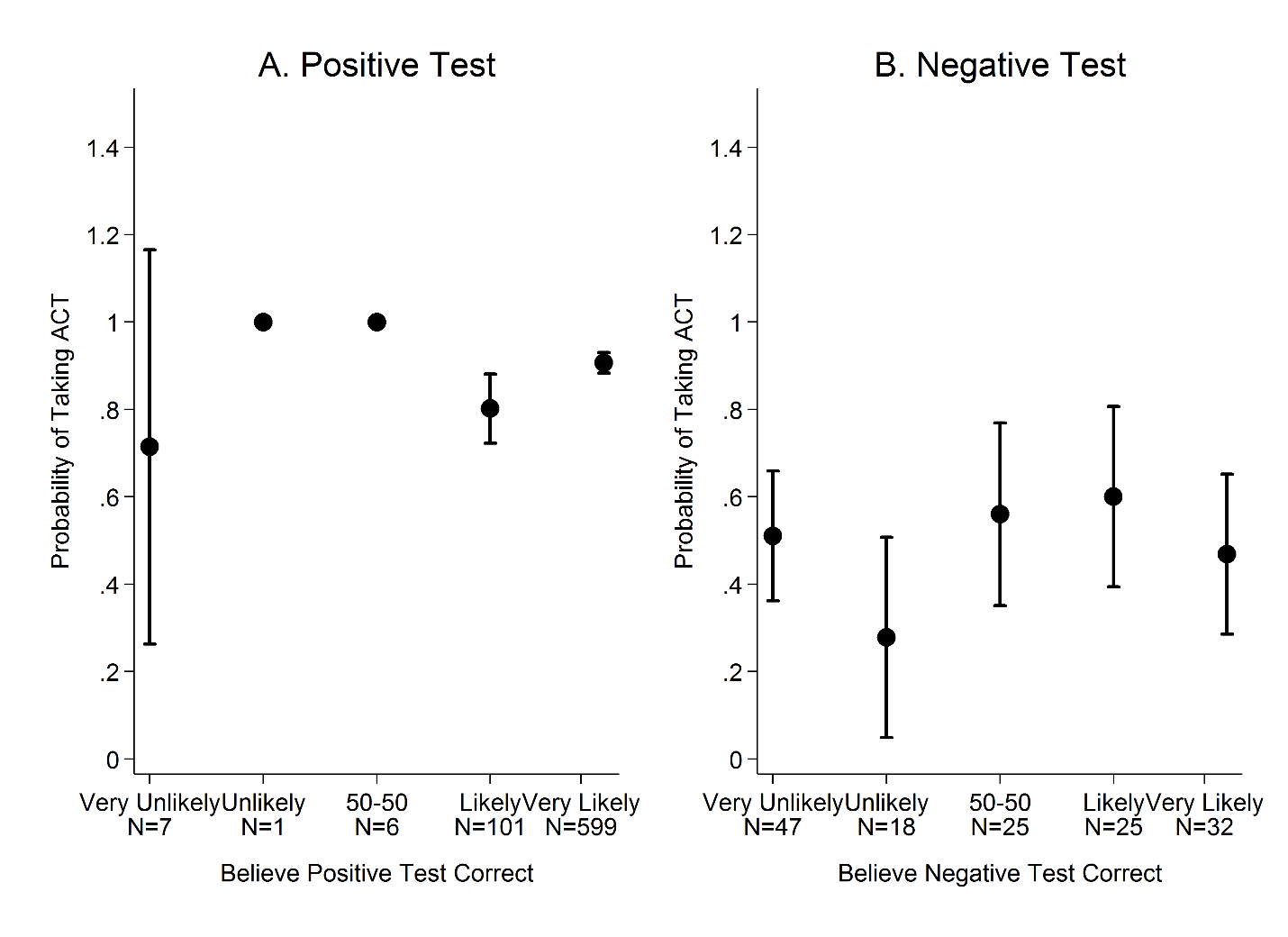


**Probability of ACT Use by Respondents’ Confidence in Testing.** Figure shows the proportion of individuals treated with an ACT separately by whether they tested positive (Panel A) or tested negative (Panel B) and by respondents’ beliefs about the likelihood that such a test result is correct. Beliefs about confidence in test were elicited *after* testing and treatment. Sample is limited to those who ever visited a health facility and were tested for malaria (N=875). 4 individuals were missing information on their test result, and an additional 6 and 4 individuals were missing information on beliefs about a positive and negative test respectively.
