# Supplementary material for: Household beliefs about malaria testing and treatment in Western Kenya: the role of health worker adherence to malaria test results
Source: Malar J. 2017 Aug 22;16:349. doi: 10.1186/s12936-017-1993-7 (PMC5568326; doi:10.1186/s12936-017-1993-7)
Supplement: Supplementary file 7 — Additional file 7. Associations between Test Status, ACT Use, and Confidence in ACT- Individuals with Test Record and ACT Packaging Only. Table shows logistic regression results of the association between test status and ACT use (Columns 1 and 2) and beliefs about ACT effectiveness (Columns 3 and 4) for individuals who had a record of their test result and ACT-takers who showed the packaging of their drug. [file 12936_2017_1993_MOESM7_ESM.docx]

**Associations between Test Status, ACT Use and Confidence in ACT- Individuals with Test Record and ACT Packaging Only**

|  | Outcome: Odds of Taking ACT | |  |  | Outcome: Respondent Believed ACT "Very Likely" Effective in Treating Malaria | |
| --- | --- | --- | --- | --- | --- | --- |
|  | OR | AOR |  |  | OR | AOR |
|  | (1) | (2) |  |  | (3) | (4) |
| A. Tested Positive for Malaria | 7.69** | 8.76** |  |  | 1.49 | 1.43 |
|  | [4.52,13.07] | [5.02,15.28] |  |  | [0.69,3.23] | [0.55,3.72] |
|  |  |  |  |  |  |  |
| B. Tested Negative for Malaria | 0.42** | 0.41** |  |  | 0.28* | 0.28 |
|  | [0.25,0.69] | [0.24,0.71] |  |  | [0.08,0.96] | [0.07,1.05] |
|  |  |  |  |  |  |  |
| C. Not Tested for Malaria | Ref. Group | Ref. Group |  |  | Ref. Group | Ref. Group |
|  |  |  |  |  |  |  |
|  |  |  |  |  |  |  |
| Includes Controls |  | X |  |  |  | X |
| Mean of Outcome in Reference Group | 0.51 | 0.51 |  |  | 0.67 | 0.67 |
| P value: (A=B) | 0 | 0 |  |  | 0.002 | 0.003 |
| Number of Obs | 414 | 407 |  |  | 269 | 267 |

Notes: Table shows logistic regression results of the association between test status and ACT use (Columns 1 and 2) and beliefs about ACT effectiveness (Columns 3 and 4). Columns 3 and 4 are limited to individuals who were treated with an ACT. The controls in Columns 2 and 4 include the following: the wealth of the household (defined as the first component from a principal component analysis of household characteristics and assets), the education level of the respondent (no education, some primary education, or some secondary education), the sick individual’s age and gender, and the time it takes for the household to travel to the nearest health facility. All coefficients are expressed in terms of odds ratios and 95 % confidence intervals are in brackets. Sample is limited to those who were not tested or who were tested and had a record of the result and ACT-takers who had the packaging for the drug (N=414). Standard errors are adjusted for clustering by community unit. *p<0.05, **p<0.01
