# Supplementary material for: Household beliefs about malaria testing and treatment in Western Kenya: the role of health worker adherence to malaria test results
Source: Malar J. 2017 Aug 22;16:349. doi: 10.1186/s12936-017-1993-7 (PMC5568326; doi:10.1186/s12936-017-1993-7)
Supplement: Supplementary file 8 — Additional file 8. Associations between Malaria Beliefs, Testing, and ACT Use - Individuals with Test Record and ACT Packaging Only. Table shows logistic regression results of the association between both test status and ACT use and beliefs about malaria likelihood for individuals who had a record for their test result and ACT-takers who showed the packaging of their drug. [file 12936_2017_1993_MOESM8_ESM.docx]

**Associations between Malaria Beliefs, Testing, and ACT Use - Individuals with Test Record and ACT Packaging Only**

|  | Outcome: Respondent Said Illness Was "Very Likely" Malaria | |
| --- | --- | --- |
|  | OR | AOR |
|  | (1) | (2) |
| A. Tested Positive for Malaria, Not Treated with ACT | 4.59** | 4.47** |
|  | [1.95,10.81] | [1.75,11.40] |
|  |  |  |
| B. Tested Negative for Malaria, Not Treated with ACT | 0.49 | 0.36* |
|  | [0.23,1.06] | [0.16,0.79] |
|  |  |  |
| C. Not Tested for Malaria, Treated with ACT | 3.18** | 3.21* |
|  | [1.33,7.59] | [1.17,8.79] |
|  |  |  |
| D. Tested Positive for Malaria, Treated with ACT | 8.61** | 8.47** |
|  | [4.41,16.81] | [4.41,16.28] |
|  |  |  |
| E. Tested Negative for Malaria, Treated with ACT | 1.06 | 1.09 |
|  | [0.41,2.77] | [0.43,2.77] |
|  |  |  |
| F. Not Tested for Malaria, Not Treated with ACT | Ref. Group | Ref. Group |
|  |  |  |
|  |  |  |
| P-value: A=D | 0.152 | 0.207 |
| P-value: B=E | 0.037 | 0.002 |
|  |  |  |
| Includes Controls |  | X |
|  |  |  |
| Mean Proportion Believed Illness "Very Likely" Malaria in Reference Group | 0.35 | 0.35 |
| Number of Obs | 402 | 395 |

Notes: Table shows logistic regression results of the association between both test status and ACT use and beliefs about malaria likelihood. The controls in Column 2 include the following: the wealth of the household (defined as the first component from a principal component analysis of household characteristics and assets), the education level of the respondent (no education, some primary education, or some secondary education), the sick individual’s age and gender, and the time it takes for the household to travel to the nearest health facility. All coefficients are expressed in terms of odds ratios and confidence intervals are in brackets. Sample is limited to those who were not tested or who were tested and had a record of the result and to ACT-takers who showed their drug package (N=414). Standard errors are adjusted for clustering by community unit. *p<0.05, **p<0.01
